# Yield of household contact investigation of patients with pulmonary tuberculosis in southern Ethiopia

**DOI:** 10.1186/s12889-020-08879-z

**Published:** 2020-05-20

**Authors:** Mubarek A. Yassin, Kesetebirhan D. Yirdaw, Daniel G. Datiko, Luis E. Cuevas, Mohammed A. Yassin

**Affiliations:** 1 TB REACH Project, Awassa, Ethiopia; 2 FHI360, Addis Ababa, Ethiopia; 3 Liverpool School of Tropical Medicine (LSTM), Liverpool, UK; 4 The Global Fund, Geneva, Switzerland

**Keywords:** Tuberculosis, Index case, Household contacts, Contact investigation, Case finding

## Abstract

**Background:**

Household Contacts (HHCs) of patients with pulmonary tuberculosis (PTB) have a higher risk of developing TB. Contact investigation is recommended to reach this group and identify undiagnosed cases. In this study, we have determined the yield of contact investigation among HHCs of patients with smear-positive PTB, and estimated TB burden.

**Methods:**

We conducted retrospective record review for the occurrence of TB among HHCs of Index PTB+ cases treated between November 2010 and April 2013 in 12 public health facilities in Boricha district. HHCs were followed up monthly and revisited between March and June 2015. Information on additional TB cases diagnosed and treated among HHCs were documented. HHCs who were diagnosed as having TB after the index cases were diagnosed and treated were considered as ‘incident cases’. Presumptive TB case was defined as those having cough for ≥2 weeks or enlarged lymph node. Diagnosis of TB among HHCs were made using smear-microscopy and/or X-rays, and clinically for Extra-pulmonary TB (EPTB).

**Results:**

One thousand five hundred and seventeenth HHCs of 344 index cases were visited and screened for TB and followed up for a median of 37 months. 77 (5.1% - 72 with PTB and 5 with EPTB) HHCs developed TB during 4713 person-years of follow-up with an estimated incidence of 1634 (95% CI: 1370-2043) per 100,000 person-years follow-up which is much higher than the estimated TB incidence for the general population in Ethiopia of 210/100,000. Half (41/77) of incident TB cases were diagnosed within the first year of diagnosis of the index cases and 88% (68/77) were adults (Hazard Ratio: 4.03; 95% CI: 2.00–8.12).

**Conclusion:**

HHCs of index PTB+ cases have high risk of developing active TB. Long term follow-up of HHCs could help improve TB case finding depending on country contexts. Further studies on effectiveness and feasibility of the approach and integration in routine settings are needed.

## Background

Tuberculosis (TB) case finding strategies mostly rely on passive case finding. According to World Health Organization (WHO) over 30% of the estimated new TB cases are missed every year due to being not diagnosed or diagnosed and treated, but not reported to national TB programs (NTP) [1]. Missed opportunities to identify these cases include case finding among contacts of index cases in the community.

WHO recommends contact investigation (CI) for household contacts (HHCs) of patients with bacteriologically confirmed TB in low and middle income countries [2]. CI identifies individuals at high risk for developing tuberculosis [3–7] and offers an opportunity for early diagnosis and treatment of contacts with active TB, to interrupt transmission and reduce morbidity and mortality in affected individuals [8, 9]. However, CI is not often conducted routinely or not conducted well [10].

In Ethiopia, yield from CI has been assessed in two different ways. The first is CI within 3 months of diagnosis of index TB cases [11]. A yield of 2500/100,000 HHCs was reported in this approach in Amhara and Oromia regions of Ethiopia where 15,527 HHCs were screened for TB. While this approach identified undiagnosed cases, some of them may be prevalent cases which were not diagnosed in a timely manner and others could be new cases. In the second approach, CI was done for household (HH) members of previously diagnosed index cases [12]. The study by Zewdu et al. in Amhara and Oromia regions was conducted from June–October 2014 and indicated that in this approach, of 272,441 close contacts of 47,021 index cases diagnosed 2 years before the study started, TB notification was 768/100,000 after contact investigation. This study demonstrated that CI could identify additional TB cases (most likely new) long after index cases were diagnosed and treated. In this study, we estimated additional TB cases diagnosed among HHCs of patients with smear positive pulmonary TB (PTB+) during long term follow-up.

## Methods

We conducted retrospective record review for the occurrence of TB among household contacts of index PTB+ cases treated between November 2010 and April 2013 in 12 public health facilities in Boricha district, Sidama Zone, Southern Ethiopia. The district has a population of 314,296 and is located 33 kms from Hawassa, which is the regional capital. The district has 12 health centers providing TB diagnosis and treatment services [13]. Each health center provides support to health posts in their catchment areas, which are run by two health extension workers (HEWs). Between 2010 and 2015, a community-based TB project implemented in the district and trained HEWs who visited all households in their villages (Kebeles) and collected sputum samples from presumptive TB cases, prepared smears, fixed slides, and sent slides to nearest health facility laboratory for staining and smear microscopy. Their supervisors facilitated transportation of smears to health facilities and delivery of results back to HEWs. Smear negative cases were referred to health facilities for further investigation including chest X-rays and clinical follow-up. Supervisors also brought anti-tuberculosis drugs to HEWs whenever a person was diagnosed with TB. HEWs supported treatment and followed up patients during treatment. HEWs with District TB supervisor conducted household visits immediately after index cases initiated treatment, registered and screened consenting HHCs for active TB and conducted contact screening and monitored adherence to Isoniazid preventive therapy (IPT) at community level. HEWs also conducted regular follow up visits to households of index cases as part of routine case finding activity to identify symptomatic cases and facilitate diagnosis. Household contacts who didn’t have TB during initial contact investigation visit were followed-up by HEWs monthly. Any person having cough for two or more weeks and/or having enlarged lymph nodes was considered as presumptive TB case [14]. Diagnosis of TB among HHCs was made using smear-microscopy and/or X-rays, and clinically for Extra-pulmonary TB (EPTB). Two sputum samples, spot and morning, were collected and light-emitting-diodes-fluorescent microscopes (LED-FM) were used for diagnosis [15]. TB cases identified among HHCs were registered and received the same treatment and followed up as the index cases.

Index PTB+ cases diagnosed between November 2010 and April 2013 were selected and their HHCs were retrospectively studied. Identification and address of the index cases were collected from TB registers of the health centers. Between March and June, 2015, households of all index cases were visited by the study team to check for the occurrence of additional TB cases among HHCs between November 2010 and April 2015 and validated the data. Trained health professionals working in the health centers and health posts collected the data at household level using pre-tested questionnaire. All household members of index TB case diagnosed between Nov 2010 and April 2013 were recorded. ‘Index TB cases’ are those who were diagnosed first and all TB cases diagnosed in a household after the diagnosis of index cases were considered as ‘TB cases among HHCs’. Household contact is defined as a person who shared the same enclosed living space for one or more nights or for frequent or extended periods during the day with the index case during the 3 months before the diagnosis of TB [16]. Data collection at the households was done from March to June 2015; household head or index case was interviewed about TB status of HHCs. Data collection was supervised on daily basis to assure data quality. Data in completed questionnaire from the households was cross-checked with TB DOTS registers in the health facilities where category of TB and exact date the HHCs started treatment were collected.

Independent variables collected included age, gender, educational status, marital status, occupation, religion, family size and treatment start date for index cases while for HHCs age, gender and treatment start date. The dependent variable was TB disease among HHCs of PTB+ index cases after treatment start by the index cases. Data were entered into Epi Info 3.5.4 and exported to and analyzed using Stata statistical software version 12. The data can be accessed in the supplementary material (Additional file 1).

HHCs diagnosed with active TB during contact investigation visits were considered as ‘incident TB cases.’ Person-years at risk of TB disease was calculated as time between date index case started treatment until date of TB diagnosis made among HHCs or for those who did not develop the disease, till last follow-up date. Household contacts that changed residence or died were excluded from the analysis. TB incidence density was calculated as the number of incident TB cases among HHCs divided by the total number of HHCs’ follow-up person-years observed (PYO). A subgroup analysis of TB incidence among HHCs of index cases diagnosed between years 2010–2011 was calculated to determine TB incidence trend among people with longer follow-up. Hazard ratios, generated using Cox regression, were used to identify determinants of incident TB. *P*-value < 0.05 was considered statistically significant. Clustering effect of data because contacts to index cases are household members was adjusted by using household ID when calculating confidence interval, hazard ratio, and *p* value.

## Result

**Fig. 1:**
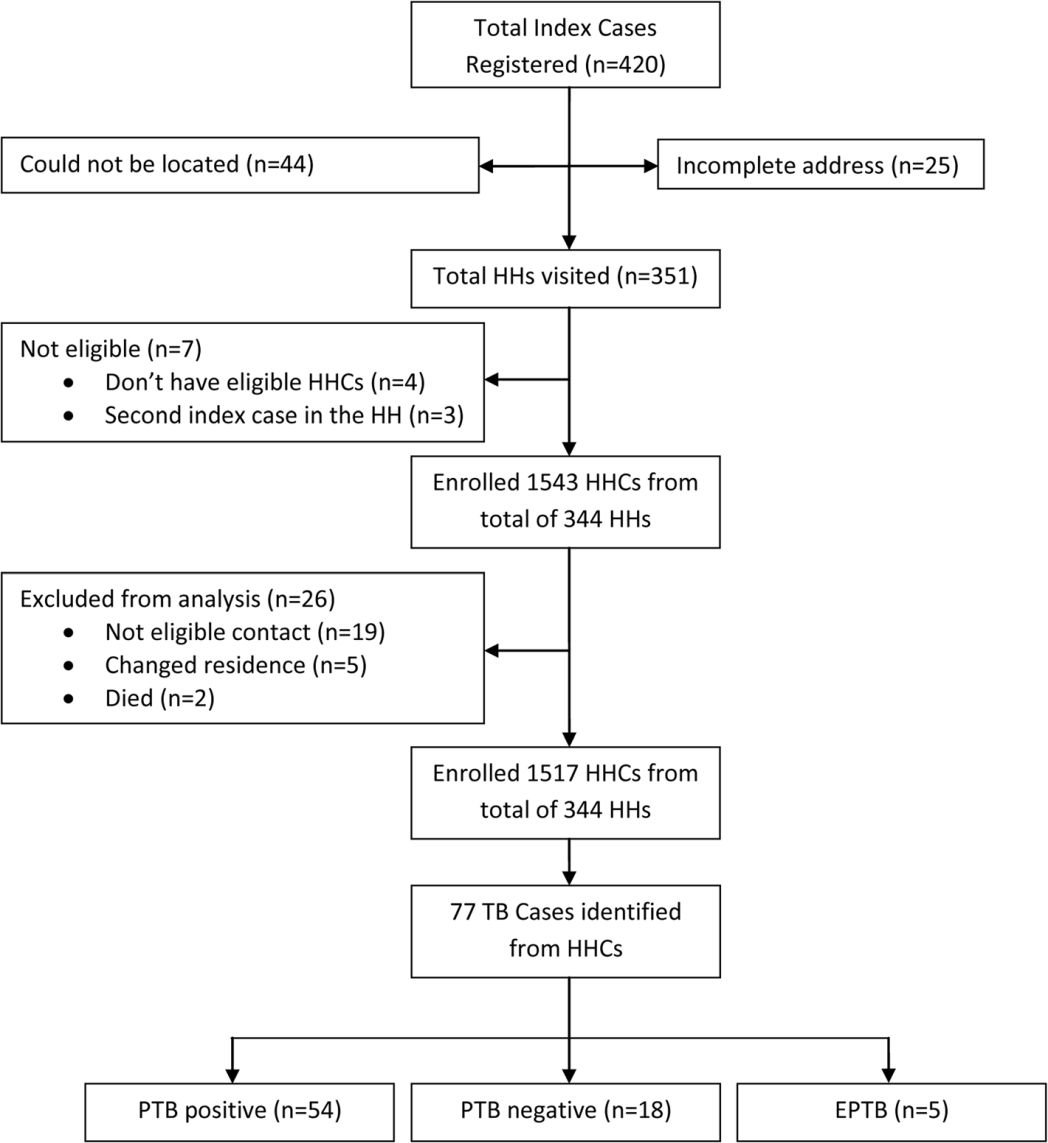
Flow chart indicating selection of study participants

Of the 420 index cases identified from TB DOTS registers from November 2010 to April 2013 for whom initial contact investigation was conducted, 44 (10%) couldn’t be located while 25 (6%) had incomplete address and were excluded. Thus, households of 351 index cases were revisited in 2015. Seven of these were excluded because there were no eligible HHCs (*n* = 4) or there was a second index case in the household (*n* = 3). From the remaining 344 index cases, 1543 HHCs were enumerated for follow-up but only 1517 were analyzed as 19 did not fulfill the definition of a HHC, five changed residence after follow-up started while two died (See Fig. 1). Of the 344 index cases, majority were adults (96.5%), 273 (79.4%) were married and 217 (63.1%) illiterate. In 215 (62.5%) of households, there were five or more people living together. Median age (inter-quartile range) of index and HHCs were 35 (26–45) and 18 (12–30) years, respectively. Men constituted slightly higher proportion of both index cases and HHCs (~ 57% for both). HHCs were followed-up for a median of 37 months (inter-quartile range: 30–47 months) (Table 1).

**Table 1:** Socio-demographic characteristics of index TB cases treated for TB between Nov 2010-Apr 2013) and relation to Incident TB among household contacts (treated for TB between Nov 2010-Apr 2015)

Index case or household characteristic	Response	# Index TB cases	# of TB cases among HHCs^a^	Person years for HHCs (PY)	TB incidence for HHCs per 100,000 PY (95% CI)	Adjusted hazard ratio^b^ (95% CI) for HHCs	*P* value^b^
**Age**	Children < 15	12 (3.49%)	4	147	2718 (1020–7242)	1	0.288
	Adults ≥15	332 (96.51%)	73	4566	1599 (1271–2011)	0.55 (0.18–1.65)	
**Gender**	Female	149 (43.31%)	34	1896	1793 (1281–2510)	1	0.619
	Male	195 (56.69%)	43	2817	1527 (1132–2058)	0.89 (0.55–1.43)	
**Marital status**	Single	71 (20.64%)	21	965	2177 (1420–3339)	1	0.207
	Married	273 (79.36%)	56	3749	1494 (1150–1941)	0.71 (0.41–1.21)	
**Education**	Illiterate	217 (63.08%)	42	3078	1365 (1009–1847)	1	0.180
	Elementary	94 (27.33%)	27	1189	2270 (1557–3311)	1.61 (0.97–2.69)	
	High school or above	33 (9.59%)	8	446	1793 (897–3585)	1.32 (0.6–2.94)	
**Occupation**	Housewife	99 (28.78%)	24	1211	1982 (1328–2957)	1	0.691
	Farmer	163 (47.38%)	34	2478	1372 (980–1920)	0.72 (0.42–1.25)	
	Daily laborer	55 (15.99%)	1	87	1152 (162–8178)	1.06 (0.53–2.88)	
	Student	6 (1.74%)	15	715	2099 (1266–3482)	0.59 (0.08–4.66)	
	Others	21 (6.1%)	3	222	1348 (435–4181)	0.72 (0.21–2.49)	
**Religion**	Christian	307 (89.24%)	72	4185	1721 (1366–2168)	1	0.591
	Muslim	33 (9.59%)	5	467	1072 (446–2575)	0.61 (0.24–1.57)	
	Others	4 (1.16%)	0	62	0	–	
**Family size**	< 5	129 (37.5%)	21	952	2207 (1439–3384)	1	0.262
	5–8	190 (55.23%)	48	3064	1567 (1181–2079)	0.71 (0.41–1.21)	
	> 8	25 (7.27%)	8	698	1146 (573–2292)	0.52 (0.22–1.24)	
**Total**		**344 (100%)**	**77**	**4713**	1634 (1307–2043)		

^a^*HHCs* Household contacts

^b^Adjusted to clustering within household

**Fig. 2:**
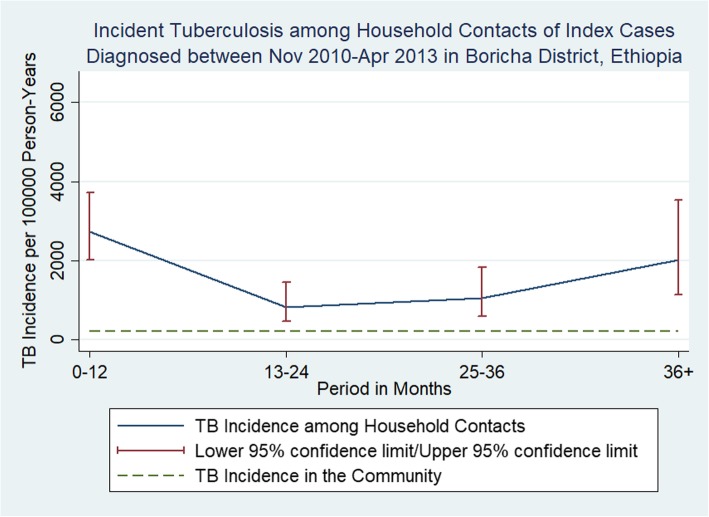
Incident Tuberculosis among Household Contacts of Index Cases Diagnosed between November 2010–April 2013

**Fig. 3:**
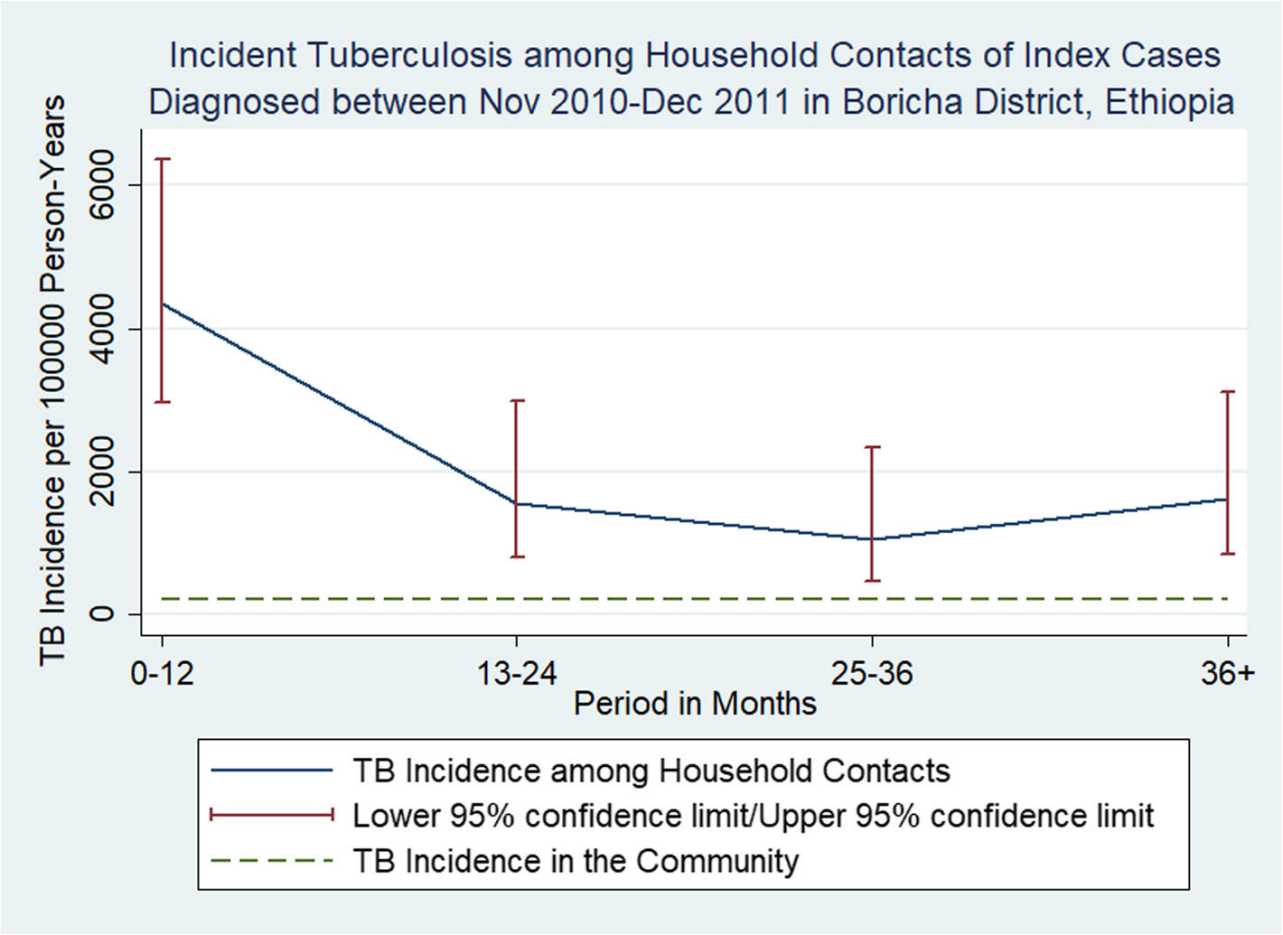
Incident Tuberculosis among Household Contacts to Index Cases Diagnosed between November 2010 and December 2011. Tuberculosis incidence was high in the first year and decreased during year two and beyond. In contrast to Fig. 2, tuberculosis incidence after year two continued to be low and the reason is that this analysis included higher proportion of household contacts that were followed for three or more years. But, note that even if TB incidence was lower for years two to four, it remained higher than that for the general population

77 (5.1%) HHCs developed TB during the 4713 person-years (PYs) of follow-up with an estimated incidence of 1634 (95% CI: 1370-2043) per 100,000 PYs. 54/77 (70.1%) had PTB+, 18/77 (23.4%) smear-negative TB and 5 had extra-pulmonary TB (Fig. 1). Majority of TB cases among HHCs were adults (68/77 = 88.3%, *p* value < 0.001). As shown in Table 2, TB incidence was highest in the first year with a magnitude of 2740 (95% CI: 2018-3772) (Figs. 2, 3).

**Table 2:** Determinants of incident tuberculosis among household contacts of index TB cases (Nov 2010-Apr 2015) in Boricha District, Sidama zone

Characteristic	Response	# TB cases among HHCs^a^	Person years	TB incidence per 100,000 (95% CI)	Hazard ratio^b^ (95% CI), adjusted	*P* value^b^
**HHC Age**	Children < 15	9	1638	549 (286–1056)	1	< 0.001
	Adults ≥15	68	3075	2211 (1743–2804)	4.03 (2.00–8.12)	
**HHC Gender**	Female	34	2124	1601 (1144–2241)	1	
	Male	43	2589	1661 (1232–2239)	0.99 (0.63–1.57)	0.971
**Time to Diagnosis**	0–12	41	1496	2740 (2018–3722)	1	0.002
	13–24	12	1469	817 (464–1439)	0.30 (0.16–0.57)	
	25–36	12	1151	1043 (592–1837)	0.38 (0.20–0.72)	
	36+	12	597	2007 (1140–3534)	0.68 (0.36–1.32)	

^a^*HHCs* Household contacts

^b^Adjusted to clustering within household

**Fig. 4:**
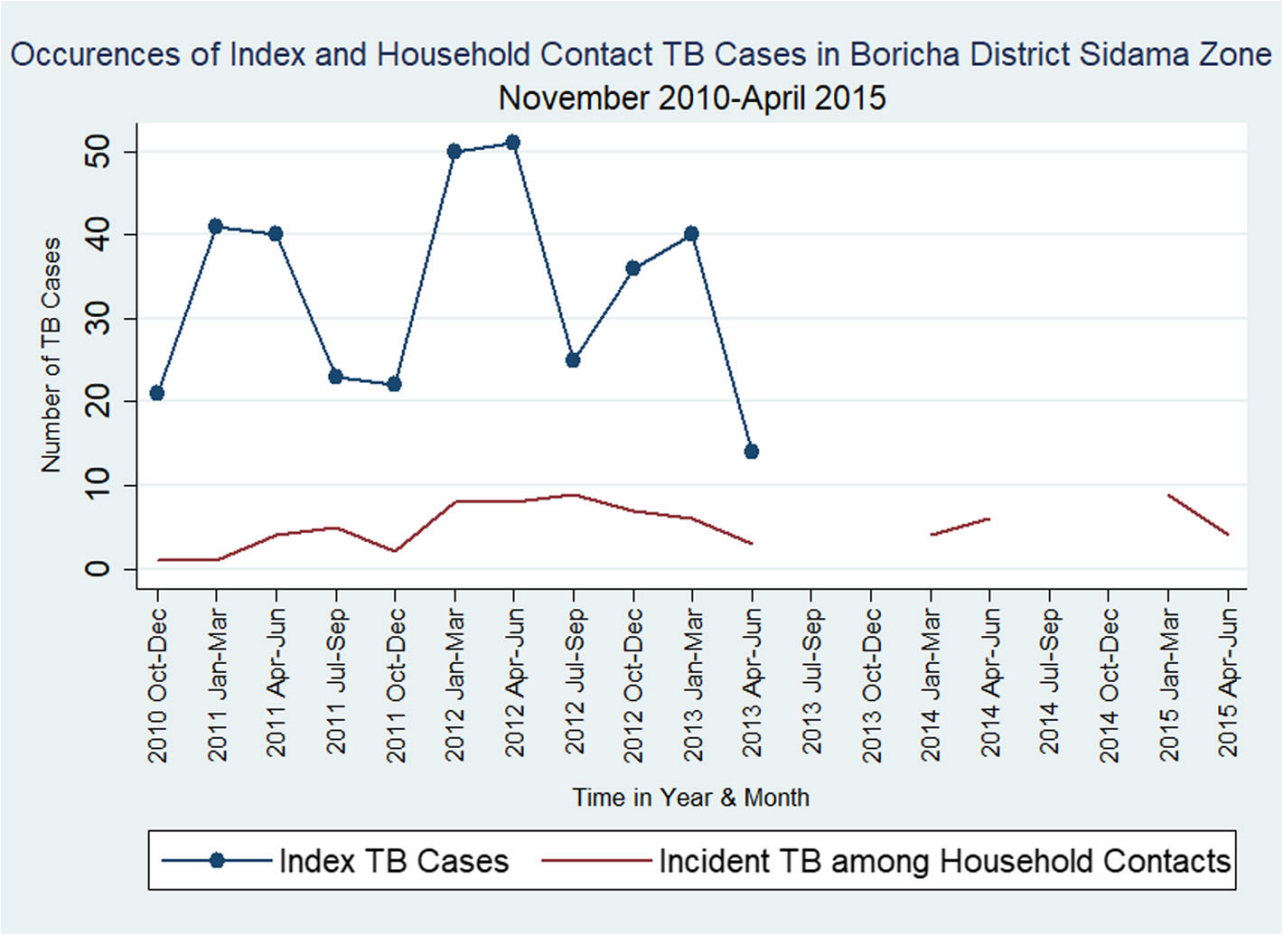
Occurrences of Index and Incident TB cases among Household Contacts across Calendar Time in Boricha District, Sidama Zone, November 2010 – April 2015

The median time between the index cases and HHCs cases diagnosed with active TB was 11 months (interquartile range: 7–29). Figure 4 indicates the distribution of index and incident TB cases among HHCs by calendar time.

## Discussion

The estimated TB burden among household contacts of index cases with PTB was high in our setting with 1634 (95% CI: 1370-2043) HHCs diagnosed with TB per 100,000 PYO. This was almost eight times higher than the estimated incidence of TB in Ethiopia for the general population which was 210 (95% confidence interval 168–250) per 100,000) in 2015 [17]. HHCs of people with PTB could develop symptoms of TB at any time and long term and regular follow up of this high risk group could be an opportunity to improve TB case finding and reach the ‘missing’ people with TB..

Overall, 77 TB cases were identified in the households of index cases at the end of follow-up period. This is 22% (77/344) or 22 TB cases identified among HHCs per 100 index PTB+ cases followed for a median of 3 years. Compared to short term prospective studies that reported 8.5% in South Africa [18] and 6.4% in Ethiopia [11], the yield from a long term follow up (average 2.2 years) was 14% in Turkey [19], 4.4% in Ethiopia [12] and 1.6% in Ghana [20] although the studies have used different approaches and duration of follow up. This indicates that long term follow-up of contacts of index cases is a potential intervention that needs consideration for implementation in routine program settings, especially in settings where community-based house-to-house visits are done as part of a routine program as it is the case in Ethiopia.

In our study, majority of TB cases among HHCs were diagnosed during the first few months after diagnosis of the index cases and then gradually declining with time. A similar trend was reported elsewhere [12, 19, 21].

In Ethiopia, there is huge potential to conduct CI and other targeted prevention interventions using the health extension worker program and health development armies [22]. Contact investigations should be intensified through active case finding and integrated with provision of preventive therapy to eligible contacts coupled with provision of education and social protection to maximize access to TB services. Sustaining household level interventions depends on cost-effectiveness. One model that was reported to be cost-effective in Vietnam for case finding was a system where the contacts were invited to come to health facility for screening and/or diagnosis than through house visits [23]. While this is cost-effective, it is likely to result in less number of household contacts visiting health facilities and getting screened as reported by another study in Ethiopia [11]. In the Vietnam study, only 2.6 contacts were screened per index case compared to 4.4 and 5.8 contacts screened in our study and the study by Gashu et al. [12] which were done through household visits. Therefore, it is important to strike the right balance between cost-effectiveness and yield of contact investigation approaches. The intensive household level follow-up could be cost effective if it is tailored to those at greatest risk of developing active TB and done through a community-based follow-up approaches [24]. Further studies are warranted to find out if facility-based and community-based contact investigation approaches could be combined to maximize cost-effectiveness with optimal yield of case finding while provision of TB preventive treatment (TPT) should be prioritized for eligible groups.

The period shortly after diagnosis of index cases and adult age were the only independent risk factors for diagnosis of TB among HHCs. The latter could be due to the difficulty of diagnosing TB among children rather than low disease incidence in this age group, as especially for under five children where obtaining appropriate specimen including through gastric lavage was not feasible in our setting where screening was conducted by HEWs [25–27]. As in our study, family size was not a determinant in another study [18]; rather it was overcrowding or close relationship that put contacts at greater risk of developing the disease [28].

Diagnostic challenges are not only for children, but also for adults. Since contact investigation is an active case finding approach, this is an opportunity for early case finding [29, 30]. While smear microscopy could be available in peripheral centers and could detect the most infectious form of TB, additional and more sensitive screening and diagnostic tests such as chest x-ray and GeneXpert are required to enable early diagnosis among high risk people such as contacts with expanded criteria for ‘presumptive TB’ than symptom-based screening.

The strength of this study comes from the fact that this was an active case finding activity that was conducted among high risk groups (HHCs). As our study is based on retrospective data, it has a number of limitations including completeness and quality of data on monthly follow-up and certain important determinants like IPT use, HIV status, malnutrition, overcrowding, and ventilation which are likely to contribute to vulnerability of HHCs [31–33]. Some indexes could not be located because of poor documentation of address and this should be taken into consideration not only when contact investigation is considered but when index cases are registered in TB DOTS registers. Reporting of episodes by HHCs and recall bias, quality of screening by HEWs, and more importantly, difficulties in categorizing cases diagnosed among HHCs as prevalent or new cases, and whether the source of infection is the index case or not. There was also no comparison group to assess if long term follow-up of HHCs resulted in identification of new cases that may not have been detected through passive case finding. As the screening and diagnostic tests used in this study were less sensitive and specific (symptom-based, clinical and smear microscopy), TB incidence is likely to be a little underestimated since GeneXpert or culture tests were not used for diagnosis.

The study was conducted in a high TB burden setting where at the time of the study TB case notification of as high as 210 per 100,000 people was reported due to implementation of innovative community-based TB care through engagement of HEWs [14]. Since contact investigation is dependent on number of index cases followed, case finding may be lower in settings where there is low index case identification. Hence, the contribution of contact investigation to case finding in routine setting where TB case notification is as low as 140 per 100,000 population could be smaller [34].

Despite all the limitations, we think that the findings could be relevant for NTPs to consider as part of their strategy/approach to find the missing people with TB depending on country contexts and availability of resources while further studies on cost effectiveness of the strategy in different programmatic and routine settings would be needed. It is also important to note that most new TB cases identified were among the general population and not among household contacts. This means interventions to reduce transmission at community level including early diagnosis, treatment and prevention are far more important to end TB by 2030 in line with the targets in the Sustainable Development Goals [35].

## Conclusion

HHCs of index TB cases remain at a higher risk of developing TB disease even after years of diagnosis of index cases. Regular follow up of HHCs is an opportunity to identify undiagnosed TB cases and should be considered as part of TB case finding strategy wherever this is feasible. To accelerate the effort to end TB, any contact investigation approach should also ensure provision of TPT for eligible HHCs.

## Supplementary information

**Additional file 1.**

## Data Availability

The dataset supporting the conclusions of this article is included within the article and its additional file (Additional file 1).

## Abbreviations

CI: Contact investigation
DOTS: Directly observed short course treatment
EPTB: Extra-pulmonary tuberculosis
HEWs: Health extension workers
HH: Household
HHCs: Household contacts
IPT: Isoniazid preventive therapy
PTB: Pulmonary TB
PTB+: Smear-positive pulmonary tuberculosis
PYO: Person-years observed
TB: Tuberculosis
WHO: World Health Organization
